# Supplementary material for: Influence of species and processing parameters on recovery and content of brain tissue-derived extracellular vesicles
Source: J Extracell Vesicles. 2020 Jun 30;9(1):1785746. doi: 10.1080/20013078.2020.1785746 (PMC7480582; doi:10.1080/20013078.2020.1785746)
Supplement: Supplemental Material [file ZJEV_A_1785746_SM2442.zip › Supplementary/figure legend.docx]

**Figure S1. Comparison of bdEVs in different fractions of SDGU and SEC: human and mouse brain.** (A) Particle concentration of SDGU fractions from human and mouse brain by NTA (Particle Metrix). (B) Particle concentration of SEC fractions from human and mouse brain (NTA). (A)-(B): Data are presented as the mean with range. (C) Three fractions from SDGU visualized by TEM (scale bar = 100 nm). (D) Fractions 7-10 from SEC (TEM, scale bar = 100 nm). TEM is representative of five images taken of each fraction from three independent brain tissue samples. (E) Western blot analysis of calnexin, CD63, TSG101, CD81, and CD9 of brain tissue and SDGU fractions (human) (n=1). (F) Western blot analysis of GM130, calnexin, CD63, syntenin, and CD81 of brain tissue and SEC fractions (human) (n=1). (G) Western blot analysis of GM130, calnexin, Bip, TSG101, syntenin, CD9, and CD81 of brain tissue and SDGU fractions (mouse) (n=1). (H) Western blot analysis of Bip and Rab27a of brain tissue and SEC fractions (mouse) (n=1).

**Figure S2. Western blot analysis: mouse brain tissue and EV preparations.** In each of the following, tissue proteins were compared with EV fractions obtained as indicated. (A) SDGU fractions; immunoblotting for GM130, calnexin, Bip, and TSG101 (n=3). (B) SEC + SDGU and SEC + UF fractions; immunoblotting for calnexin, Bip, and TSG101 (n=1). (C) SEC + UF method fractions; immunoblotting for calnexin, Bip, CD9, and TSG101 (n=2). (D) SEC + UC; immunoblotting for GM130, calnexin, and TSG101 (n=1).

**Figure S3. Bioanalyzer analysis of bdEV small RNA libraries.**Size distribution of bdEV libraries from 2H, 6H, and 24H postmortem interval (PMI) macaque brains (n=2), measured by Bioanalyzer.

**Figure S4. Effect of postmortem interval on bdEV protein contents.**(A) Venn diagram of identified protein number of BH, 10K, and EVs  at 2H, 6H, and 24H PMI in either two macaques. (B) Enrichment of cellular compartments of proteins identified in 10K and EVs  at 2H, 6H, and 24H PMI. Gene ontology (GO) analysis was performed with Funrich. GO terms with FDR-corrected p-value < 0.05 are shown). (C) Western blot analysis of calnexin, CD63, CD81, TSG101, and syntenin associated with BH and EVs of macaque brain tissue at different PMI. Blots are representative of two independent tissue EV separations.

**Figure S5. Size distribution of bdEVs from macaque and human brain as determined by nanoFCM.** The size distribution for bdEV separated by SEC+UC from human and macaque was measured by nanoFCM flow nano-Analyzer (NanoFCM Co.). Data are presented as mean with standard deviation (n=2 for macaque, n=7 for human).

**Figure S6. Enrichment of cellular and extracellular vesicle marker proteins in human brain tissue and EVs.** Log_2_ fold-change (Log_2_FC) of cellular proteins enriched in BH compared with 10K and EVs (left panel) and extracellular proteins enriched in EVs compared with 10K and BH (right panel) (n=7).
