# Supplementary figures and images for: Influence of species and processing parameters on recovery and content of brain tissue-derived extracellular vesicles
Source: J Extracell Vesicles. 2020 Jun 30;9(1):1785746. doi: 10.1080/20013078.2020.1785746 (PMC7480582; doi:10.1080/20013078.2020.1785746)

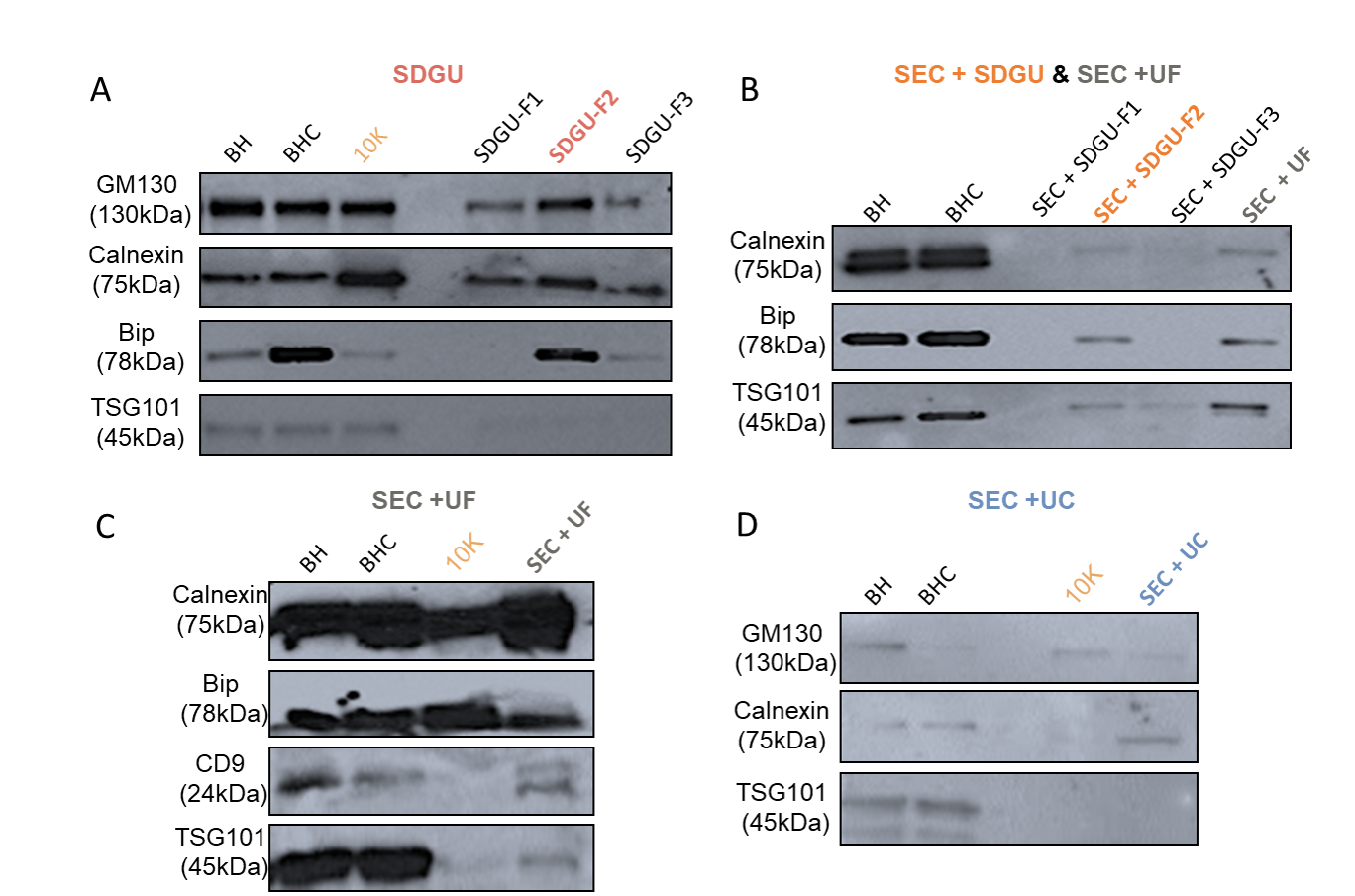

Supplement: Supplemental Material [file ZJEV_A_1785746_SM2442.zip › Supplementary/S2.tif]

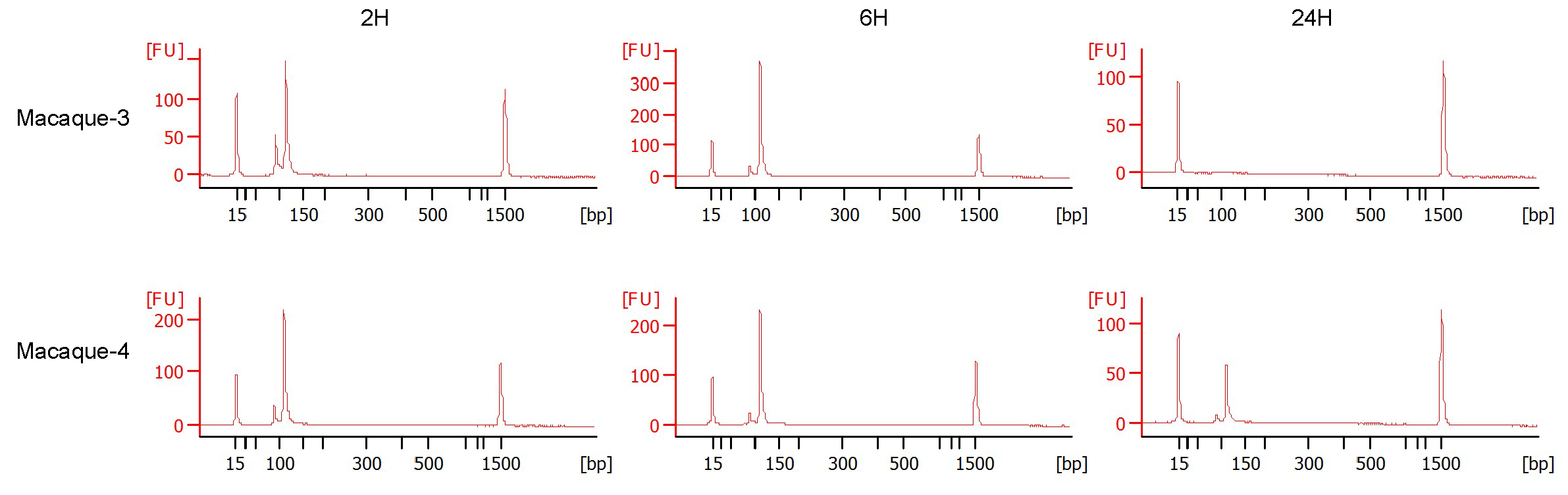

Supplement: Supplemental Material [file ZJEV_A_1785746_SM2442.zip › Supplementary/S3.tif]

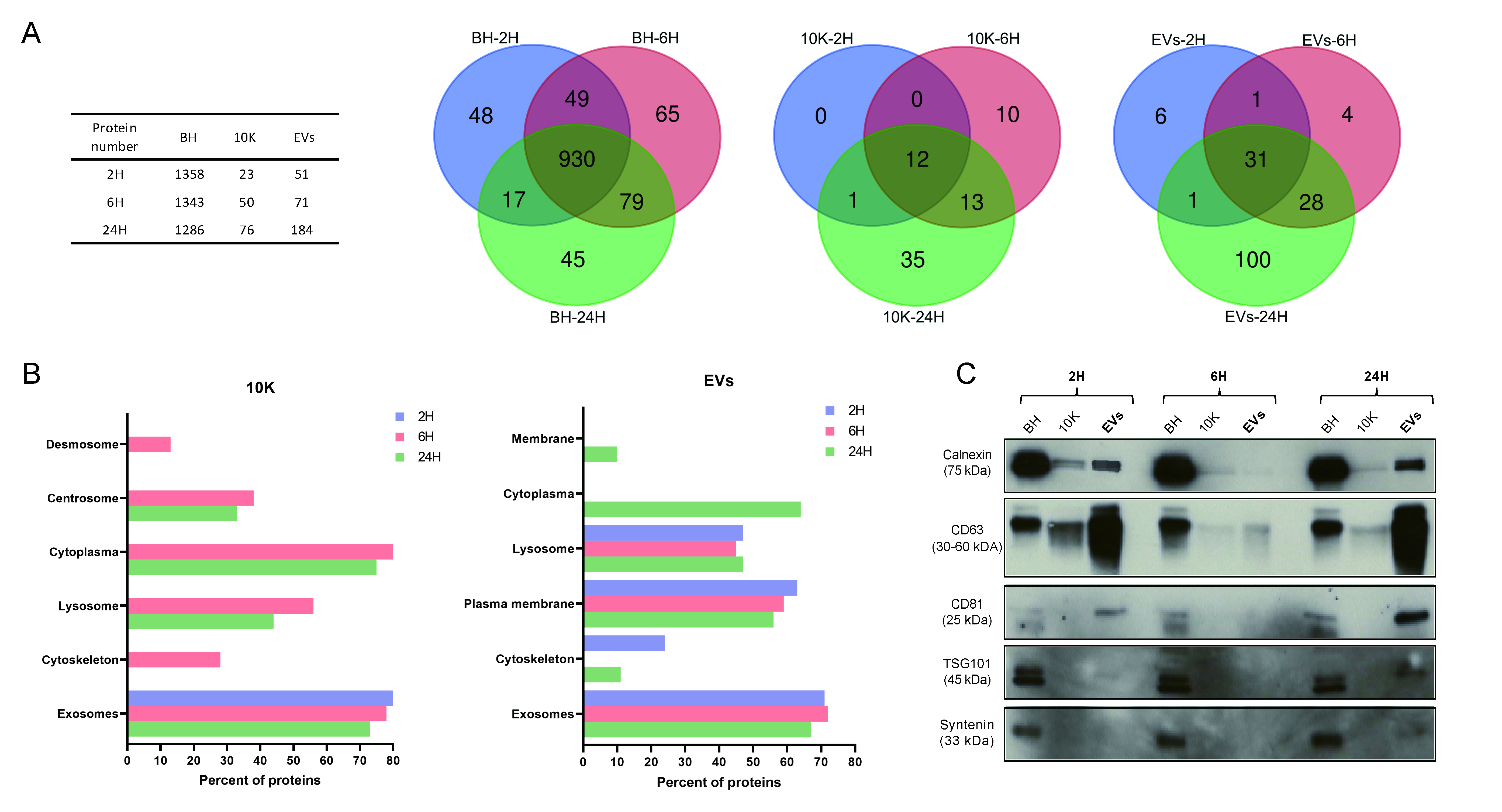

Supplement: Supplemental Material [file ZJEV_A_1785746_SM2442.zip › Supplementary/S4.tif]

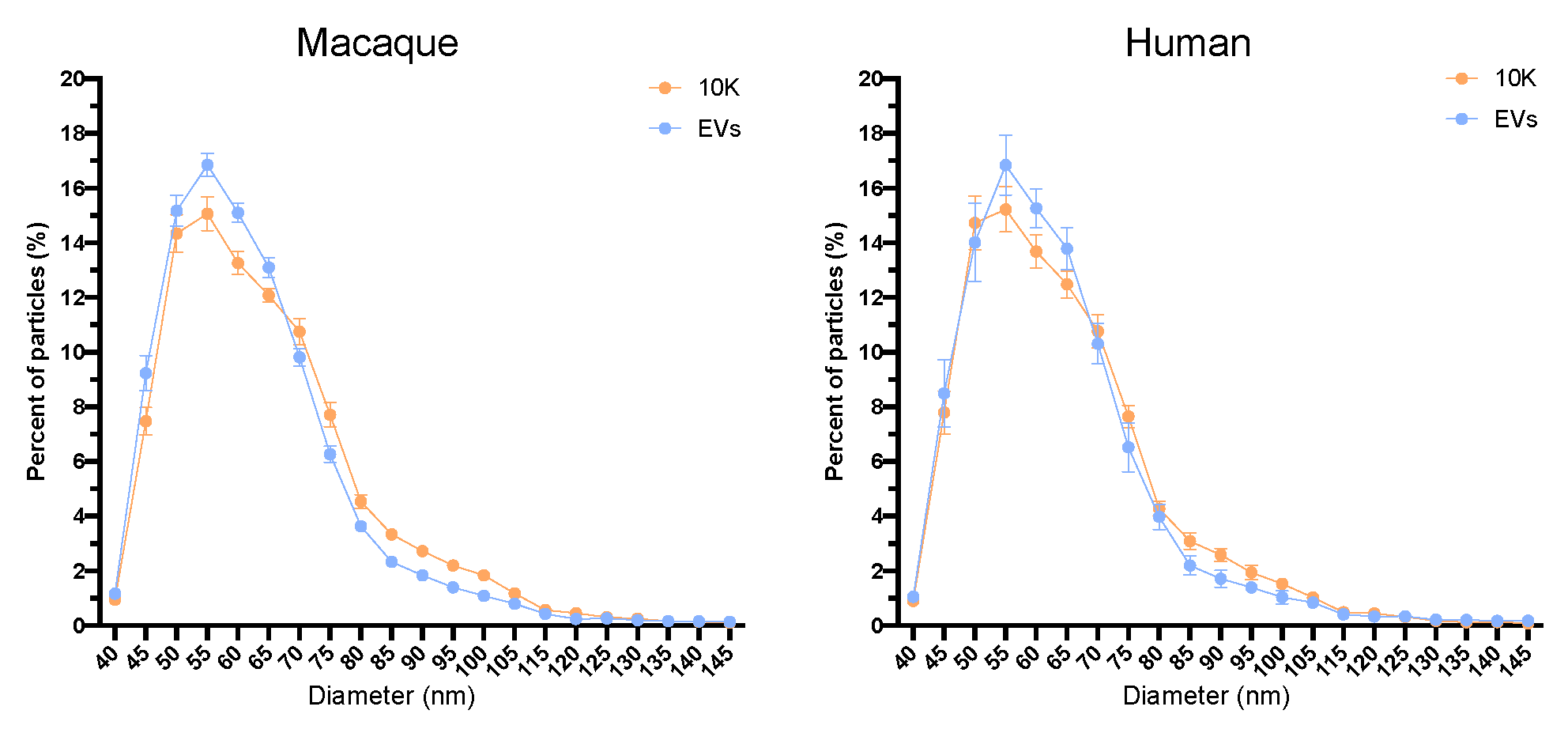

Supplement: Supplemental Material [file ZJEV_A_1785746_SM2442.zip › Supplementary/S5-Human and mouse bdEV size.tif]

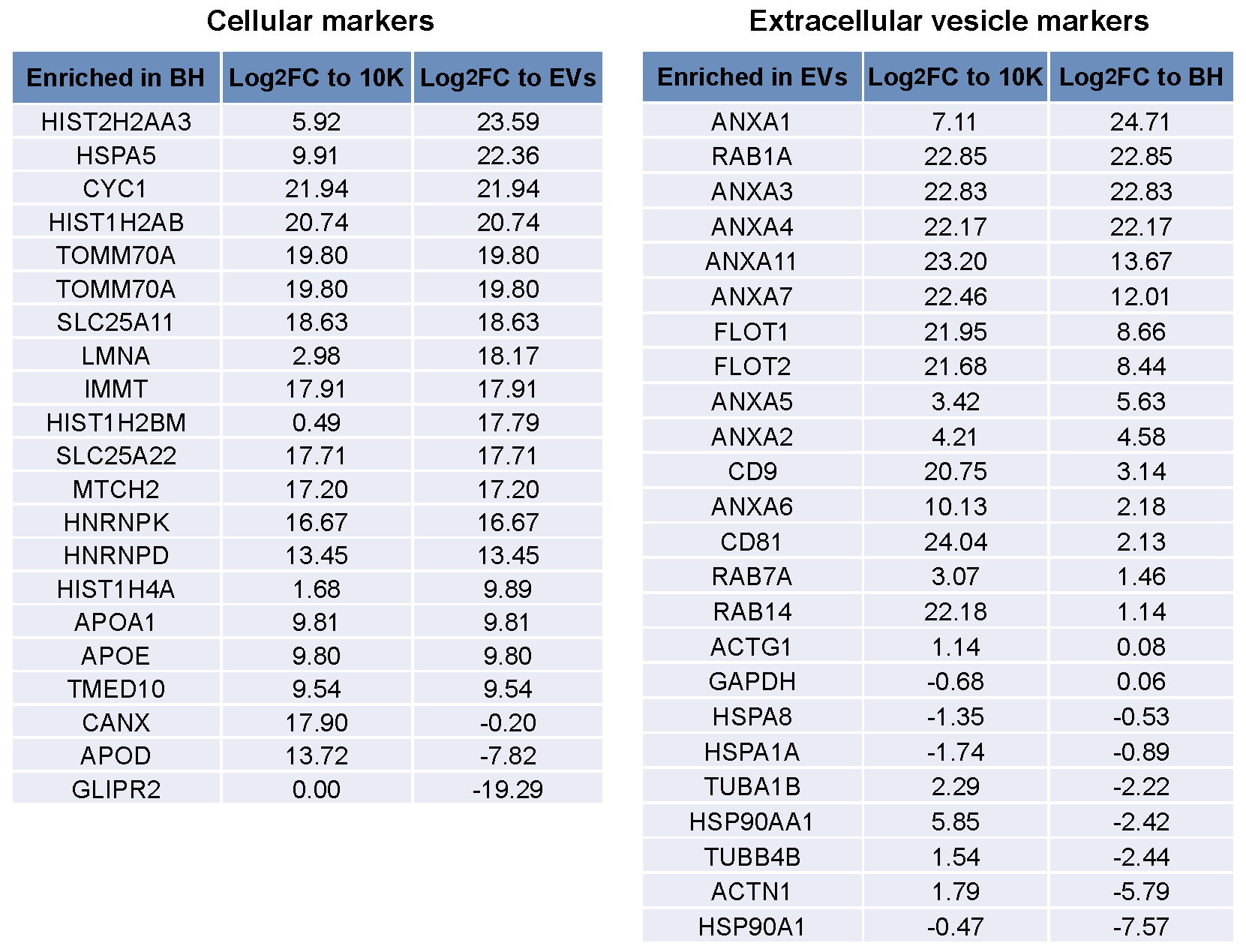

Supplement: Supplemental Material [file ZJEV_A_1785746_SM2442.zip › Supplementary/S6-EV and cell markers.tif]
